# MARCKS regulates neuritogenesis and interacts with a CDC42 signaling network

**DOI:** 10.1038/s41598-018-31578-0

**Published:** 2018-09-05

**Authors:** J. J. Brudvig, J. T. Cain, R. M. Sears, G. G. Schmidt-Grimminger, E. S. Wittchen, K. B. Adler, H. T. Ghashghaei, J. M. Weimer

**Affiliations:** 1 Pediatrics and Rare Diseases Group, Sanford Research, Sioux Falls, SD 57104 USA; 2 Basic Biomedical Sciences, University of South Dakota Sanford School of Medicine, Vermillion, SD 57069 USA; 3 Department of Cell Biology & Physiology, University of North Carolina, Chapel Hill, NC 27599 USA; 4 Department of Pediatrics, University of South Dakota, Sioux Falls, SD 57105 USA; 5 Molecular Biomedical Sciences, College of Veterinary Medicine, North Carolina State University, Raleigh, NC 27606 USA

## Abstract

Through the process of neuronal differentiation, newly born neurons change from simple, spherical cells to complex, sprawling cells with many highly branched processes. One of the first stages in this process is neurite initiation, wherein cytoskeletal modifications facilitate membrane protrusion and extension from the cell body. Hundreds of actin modulators and microtubule-binding proteins are known to be involved in this process, but relatively little is known about how upstream regulators bring these complex networks together at discrete locations to produce neurites. Here, we show that Myristoylated alanine-rich C kinase substrate (MARCKS) participates in this process. *Marcks*^*−/−*^ cortical neurons extend fewer neurites and have less complex neurite arborization patterns. We use an *in vitro* proteomics screen to identify MARCKS interactors in developing neurites and characterize an interaction between MARCKS and a CDC42-centered network. While the presence of MARCKS does not affect whole brain levels of activated or total CDC42, we propose that MARCKS is uniquely positioned to regulate CDC42 localization and interactions within specialized cellular compartments, such as nascent neurites.

## Introduction

An essential step in nervous system development is the differentiation of neurons. Neurons begin their lives as relatively spherical cells and in the hours, days and weeks after they are born, cellular machinery is reorganized to facilitate a highly complex, sprawling morphology, often with many elaborate processes. Early in this process, multiple protrusions extend from the cell body to form the primary neurites, which later extend and branch to establish mature neuronal arborization. This initial establishment of neurites is a process dependent upon the coordinated reorganization of actin filaments and bundling of microtubules at discrete locations at the membrane. Hundreds of actin modulators and microtubule-binding proteins are involved, but relatively little is known about how upstream regulators bring these polarized complexes together at discrete locations to produce neurites.

Neurite establishment utilizes many of the same proteins that are involved in polarity establishment in other nervous system cell types and at other locations within developing neurons. Cell division cycle 42 (CDC42), which regulates radial glial polarity, axonal growth cone navigation and neurite initiation, is one such example^1–3^. In nerve growth factor (NGF)-treated PC12 cells, a widely used model for neurite outgrowth, NGF treatment initiates repetitive cycles of CDC42 and RAC1 activation and deactivation, first at the cell periphery and then in the motile tips of filopodia which become neurites^4^. Importantly, both peripheral localization and repetitive cycling are necessary for neurite initiation, as overexpression of constitutively active CDC42 inhibits neurite initiation^4,5^. Driving activity of endogenous CDC42 with upstream activators, however, is sufficient to induce neuritogenesis^6^. While the importance of CDC42 for neurite initiation is well established, it is unknown what upstream regulators are responsible for the localization of CDC42 and related proteins to the specific membrane microdomains where it exerts its influence.

In the present study, we asked whether Myristoylated alanine-rich C kinase substrate (MARCKS), which is emerging as a master regulator of polarized signaling networks at the cell membrane, could have roles in neurite initiation. MARCKS is critical for the maintenance of apical polarity in radial glia, the establishment of axon guidance signaling networks and dendritic spine dynamics, all processes which utilize machinery with well-established roles in neuritogenesis^7–9^. Furthermore, MARCKS is essential for CDC42 apical membrane localization in radial glia^7^, CDC42 and RAC1 activation in human coronary artery smooth muscle cells^10^ and the distal-neurite targeting of other small GTPases in cortical neurons^11^, suggesting that MARCKS could be an important regulator of signaling networks regulating neuritogenesis.

In this study, we utilized cultured primary cortical neurons to explore roles for MARCKS in neuritogenesis and found that MARCKS is required for normal levels of primary neurite development. To begin to elucidate how MARCKS could be influencing neurite dynamics, we performed a proximity-dependent biotinylation screen to identify MARCKS interactions that are enriched in developing neurites. We identified several novel MARCKS-proximal signaling networks, including a neurite-enriched network centered around CDC42. While MARCKS does not appear to regulate whole-brain levels of activated CDC42, the two proteins stably interact, suggesting that MARCKS might regulate CDC42 targeting or scaffolding with other partners. This work introduces new functional roles for MARCKS in developing neurons, as well as new interactions which may prove critical for MARCKS-dependent neuritogenesis.

## Results

### MARCKS regulates neuronal process morphogenesis

**Figure 1:**
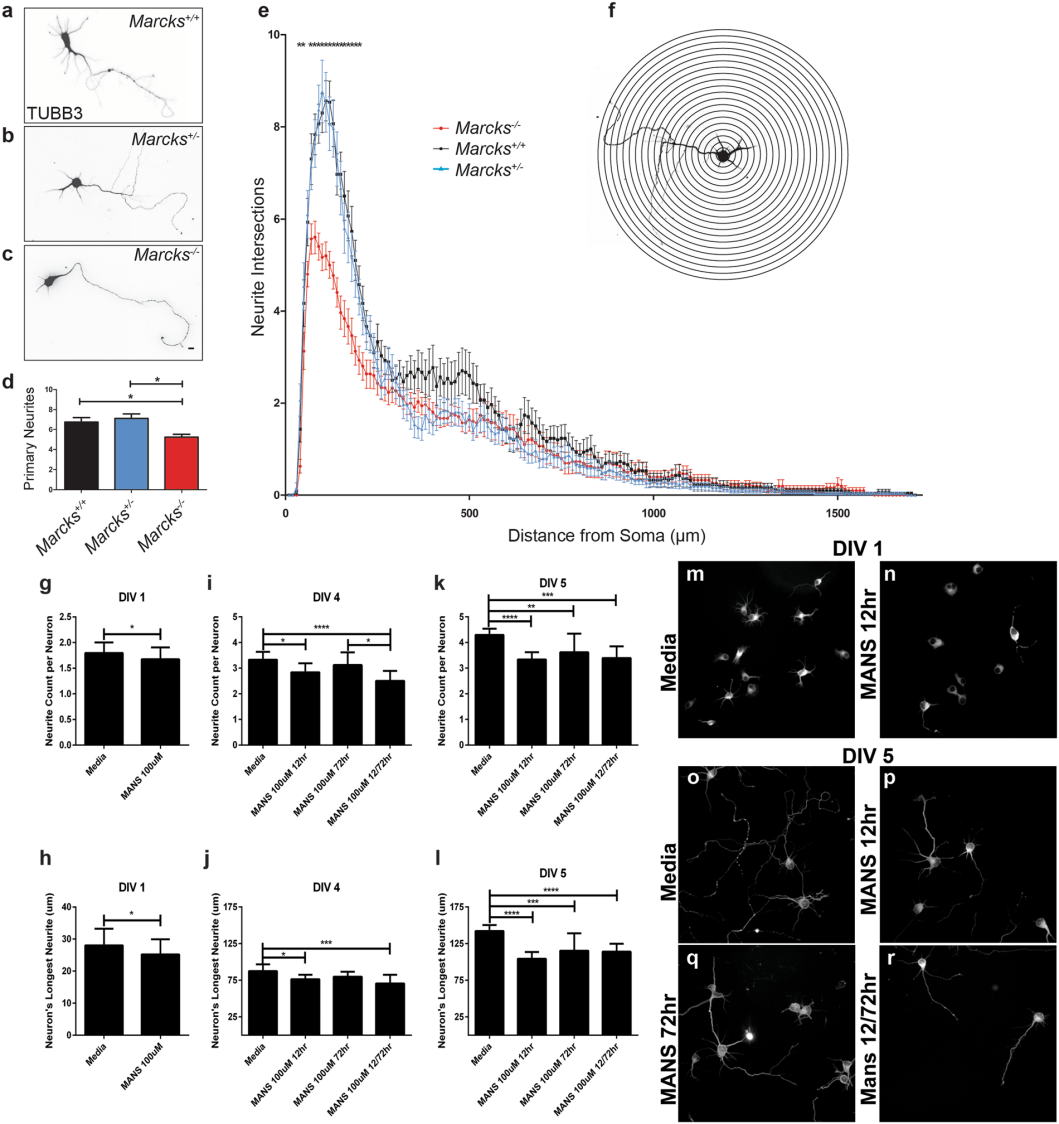
MARCKS is required for appropriate primary neurite number and neurite arborization. Primary cortical neurons were isolated from *Marcks*^+/+^, *Marcks*^−/+^ and *Marcks*^*−/−*^ mouse embryos and grown for 5 DIV in 2-dimensional culture. Neuronal somas and neurites were visualized with anti-TUBB3 antibodies (**a**–**c**) and arborization was mapped and quantified with Sholl analysis (**e**), which is depicted in a simplified cartoon in (**f**). *Marcks*^*−/−*^ neurons formed significantly fewer primary neurites (**d**) and had significantly less complex arbors (**e**) than *Marcks*^+/+^ or *Marcks*^−/+^ neurons, which were indistinguishable. One-way ANOVA, *p < 0.05 level using post-hoc Tukey’s correction for multiple comparisons. n = 30 (neurons/genotype). High Content Screening was used to quantify neurites per neuron (**g**,**i** and **k**) and measure longest neurite on a neuron (**h**,**j** and **l**) at DIV 1 (**g** and **h**), DIV 4 (**i** and **j**) and DIV 5 (**k** and **l**) in the presence of media only or 100 uM of MANS peptide administered at either 12 hours, 72 hours, or 12 and 72 hours. Bar graphs represent the mean (n = 8–12 wells per condition, with an average of 150 neurons samples per well) with the standard deviation for error bars. Representative images from the High Content Screening showing DIV 1 neurons in media only (**m**) or with 100 uM MANS peptide added after 12 hours and DIV 5 neurons administered with either media only (**o**), 100 uM MANS at 12 hours (**p**), at 72 hours (**r**), or at 12 and 72 hours (**s**). Scale bar in (**c**) is 100 μm. Bar graph and Sholl plot are mean ± SEM.

While prior studies have demonstrated roles for MARCKS in dendritic spine dynamics^8^ and axon outgrowth and morphology^9,11^, roles for MARCKS in neurite initiation and dendritic arborization have not been examined in detail. We isolated *Marcks*^+/+^, *Marcks*^−/+^ and *Marcks*^−/−^ cortical neurons from E15.5 mouse embryos and grew them in two-dimensional culture on glass coverslips (Fig. 1). After 5 days *in vitro* (5 DIV), most cultured neurons extended multiple elaborated processes. We fluorescently labeled Beta-III Tubulin in order to visualize cell bodies and neurites (Fig. 1a–c) and performed Scholl analysis to quantify neurite arborization (Fig. 1e). At distances of 30 μm to approximately 250 μm from the cell body, *Marcks*^+/+^ and *Marcks*^−/+^ neurons had indistinguishable neuritic arbors, while *Marcks*^−/−^ neurons had significantly less complex arborization. To determine whether these differences could be due to changes in neurite initiation rather than branching, we counted primary neurites and found that *Marcks*^*−/−*^ neurons had significantly fewer primary neurites (Fig. 1d). Neurite number decreased from 6.77 ± 0.44 (Mean ± SEM) neurites in *Marcks*^+/+^ neurons and 7.13 ± 0.47 neurites in *Marcks*^+/−^ neurons to 5.23 ± 0.28 neurites in *Marcks*^*−/−*^ neurons. This change was largely responsible for the decreased arbor complexity detected by Sholl analysis.

MANS peptide is a potent inhibitor of MARCKS function^49–54^. Neurons collected from E15.5 wild type mice were treated with the MANS peptide (100 uM) at 12, 72, or 12 and 72 hours after initial plating. Neurons were fixed at DIV 1, 4 and 5 and were subsequently fluorescently labeled with Beta-III Tubulin. High content imaging was then used to quantify the number of neurites per neuron and the longest neurite length per given neuron (Fig. 1g–s). At DIV 1 MANS peptide administered at 12 hours slightly but significantly reduced the number of neurites per neuron and the average length of the longest neurite on a given neuron. At DIV 4 and 5, inhibition of MARCKS with the MANS peptide reduced neurite number and length when administered at 12 hours alone, or 12 and 72 hours. When only administered at 72 hours, there was no statistical difference when compared to control at DIV 4 and the reduction of neurite number and length at DIV 5 was statistically less significant than the 12 hour or 12 and 72 hour regimens.

Neurite initiation is highly dependent on polarized signaling networks at the plasma membrane. Specifically, the coordinated activity of the small GTPases RAC1, CDC42, RHO1 and related proteins defines sites of neurite initiation^3,12–15^. Since prior studies have demonstrated roles for MARCKS in the establishment and maintenance of polarized signaling networks at the plasma membrane^7,9^, we asked whether MARCKS could be interacting with protein complexes known to mediate neurite initiation.

### MARCKS participates in protein complexes regulating neurite initiation and outgrowth

**Figure 2:**
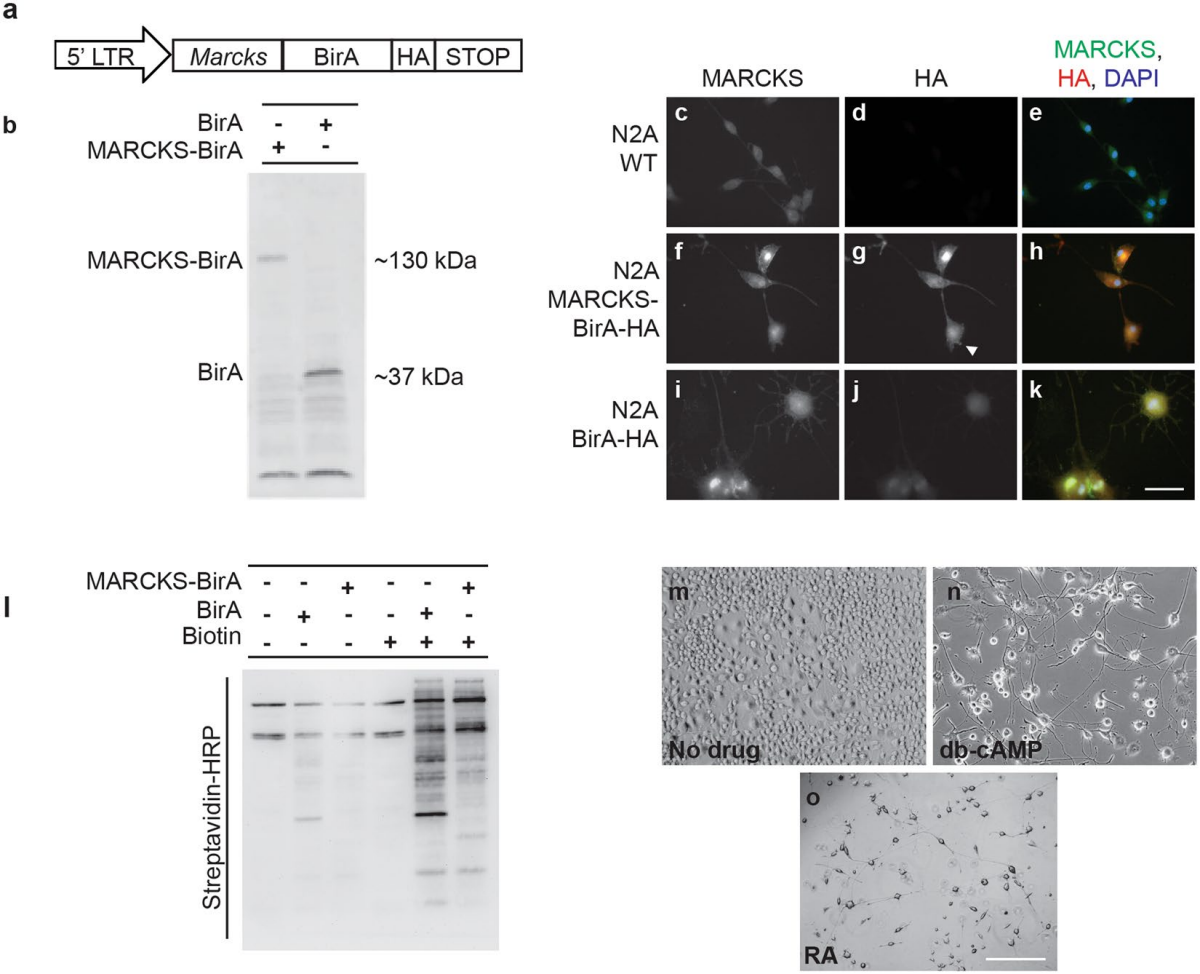
MARCKS-BirA localizes to the same cellular compartments as MARCKS and biotinylates proximal proteins *in vitro*. (**a**) Schematic of the MARCKS BioID expression construct for generation of N2A stable cell lines. (**b**) MARCKS-BirA and BirA control constructs were expressed at the predicted size. (**c**–**k**) Immunofluorescent visualization of total MARCKS (**c**,**f**,**i**) and MARCKS-BirA-HA (**g**) or HA-tagged BirA (**j**). MARCKS-BirA-HA localizes throughout the cytosol with enrichment at the plasma membrane (white arrowhead in g). (**l**) MARCKS-BirA stable cells and BirA only control stable cells biotinylate different sets of proteins in N2A cells. WT N2A and stable cell lines were grown to ~80% confluency and treated with 50 uM biotin in media or with control media for 24 hours before lysis and Western blotting. Blot is probed with HRP-streptavidin to visualize biotinylated proteins. (**m**–**o**) While untreated N2A cells are largely spherical and undifferentiated (**m**), cells treated with db-cAMP (**n**) or RA (**o**) extend multiple neurites. Scale bar in (**k**) is 100 μm and scale bar in (**o**) is 1000 μm.

To identify MARCKS-associated protein complexes involved in process initiation, outgrowth and branching, we utilized BioID, a proximity-dependent biotinylation technique that allows for the capture and identification of proximal interacting proteins^16–18^. For our BioID screen, we used the Neuro2A (N2A) mouse neuroblastoma cell line, which can differentiate with neuron-like morphology when induced with various drugs. Undifferentiated cells are largely compact, with few processes, while differentiated cells extend many processes with various degrees of branching. By exploring the unique repertoire of MARCKS interactors in differentiated cells, we hoped to identify MARCKS-associated pathways and protein complexes involved in neurite morphogenesis. We created Neuro2A stable cell lines^19^ expressing a MARCKS-BirA-HA fusion transgene under control of the 5’LTR, which serves as a low level constitutive promoter (Fig. 2a). We also created control Neuro2A stable cell lines, which express only BirA-HA. Both transgenes were produced at the expected size (Fig. 2b). MARCKS-BirA-HA localized to the same cellular compartments as MARCKS, with apparent cytosolic and membrane localization (Fig. 2f–h), while BirA-HA appeared to be exclusively cytosolic (Fig. 2i–k). Both transgenes robustly biotinylated proximal proteins in the presence of exogenous biotin (Fig. 2l). We then isolated and identified biotinylated targets from undifferentiated cells (Fig. 2m) and from cells that were treated with either dibutyryl-cyclic AMP (db-CAMP, Fig. 2n), which induces formation of axon-like processes, or retinoic acid (RA, Fig. 2o), which induces formation of dendrite-like processes^20^.

**Figure 3:**
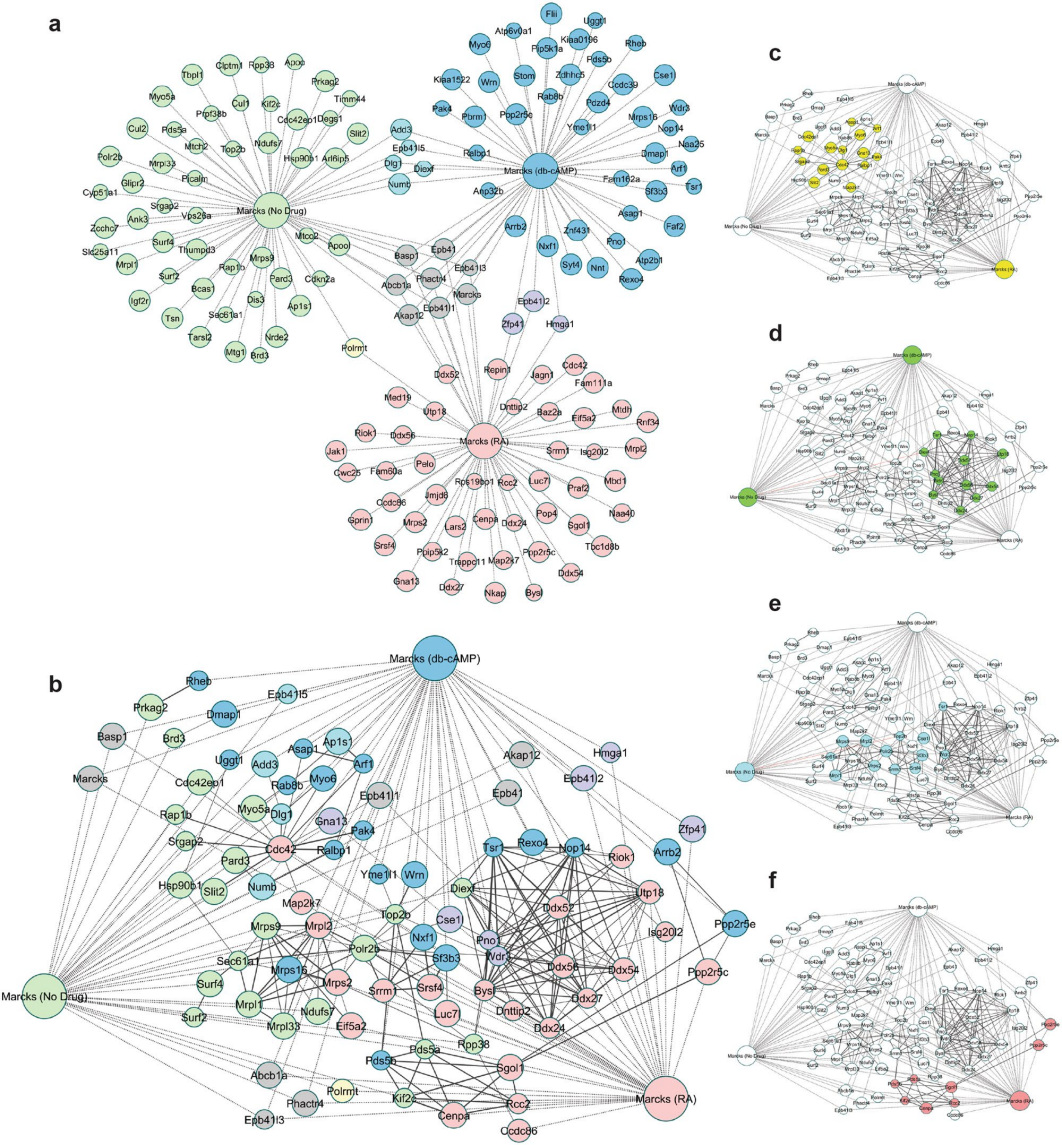
Candidate MARCKS interactors identified with MARCKS BioID. (**a**) Using BioID, MARCKS interactors were identified in untreated (green), db-cAMP treated (blue) and RA treated (red) N2A cells. Eight proteins (grey) were identified above background levels in all three conditions and several were identified in two of three conditions (yellow, teal and purple). (**b**) When interactions identified by MARCKS BioID (dotted lines) are overlaid with known interactions from the STRING database (solid lines), several putative protein complexes and signaling networks emerge. (**c**) A signaling network centered around CDC42 and cytoskeletal modulators was identified, with members in each of the three groups. (**d**) A complex comprised primarily of RNA processing proteins and DEAD box helicases was identified (**e**), as was a complex enriched for proteins involved in nuclear export, including Csel1. (**f)** An additional complex was enriched for proteins involved in control of the cell cycle and included SGOL1 and CENPA. Weight of solid lines reflects confidence of STRING associations and node size reflects fold-change of interactor vs. control.

In all, we identified 156 unique interactors, with a modest level of overlap between treatment states (Fig. 3a). There was a core group of 8 MARCKS interactors that were detected in all three groups. Interestingly, this shared group was enriched for proteins with a MARCKS ED-like domain, including AKAP12 and BASP1, suggesting that MARCKS interacts with homologous proteins regardless of cell differentiation state or cell cycle status. We also identified interactions with RALBP1 and ADD3, which also contain MARCKS ED-like domains (Supp. Fig. 1). This domain has both a PIP2-binding motif ^21,22^ and a nuclear translocation signal^22^, suggesting that MARCKS could potentially interact with these proteins to influence PIP2-related events, perhaps in the nucleus, an additional compartment where MARCKS is found^22^.

To identify potential functional relationships and protein complexes in our list of candidate interactors, we merged our data set with a list of known protein-protein interactions from the STRING database using a moderate confidence cutoff of 0.6, knowing that spurious interactions would be filtered with subsequent analysis. This generated a protein network with several obvious hubs (Fig. 3a). In order to identify high-confidence protein complexes within this network, we used a filtering algorithm to remove noise and then applied a weighted clustering coefficient^23^ to identify putative complexes (Fig. 3b). This strategy identified a number of protein complexes in which MARCKS potentially participates. Several of these complexes were specific to one differentiation state, while others were more general, clustering with multiple differentiation states.

Complex 1 was comprised of proteins associated with all three groups, but was centered around CDC42 (identified in RA treated cells) and a network of its known interactors, including small GTPases like SRGAP2 and RAP1B (Fig. 3c). This complex was of great interest to our study, considering that CDC42 and related GTPases serve critical roles in neurons, which depend on dynamic regulation of the actin cytoskeleton to establish, maintain and modify cellular processes. Additionally, members of this complex were detected predominantly in differentiated cells, which had many neurites with varying levels of extension and arborization. The largest remaining complexes consisted mainly of nuclear proteins involved in gene expression and nuclear transport, suggesting potential roles for MARCKS in the nucleus. Complex 2 consisted of a number of DEAD-box nucleic acid helicases, along with several nuclear RNA processing proteins (Fig. 3d). Complex 3 was enriched for proteins involved in nuclear export, including CSEL1 (Fig. 3e). Complex 4 was enriched for proteins involved in control of the cell cycle and included SGOL1 and CENPA (Fig. 3f).

**Figure 4:**
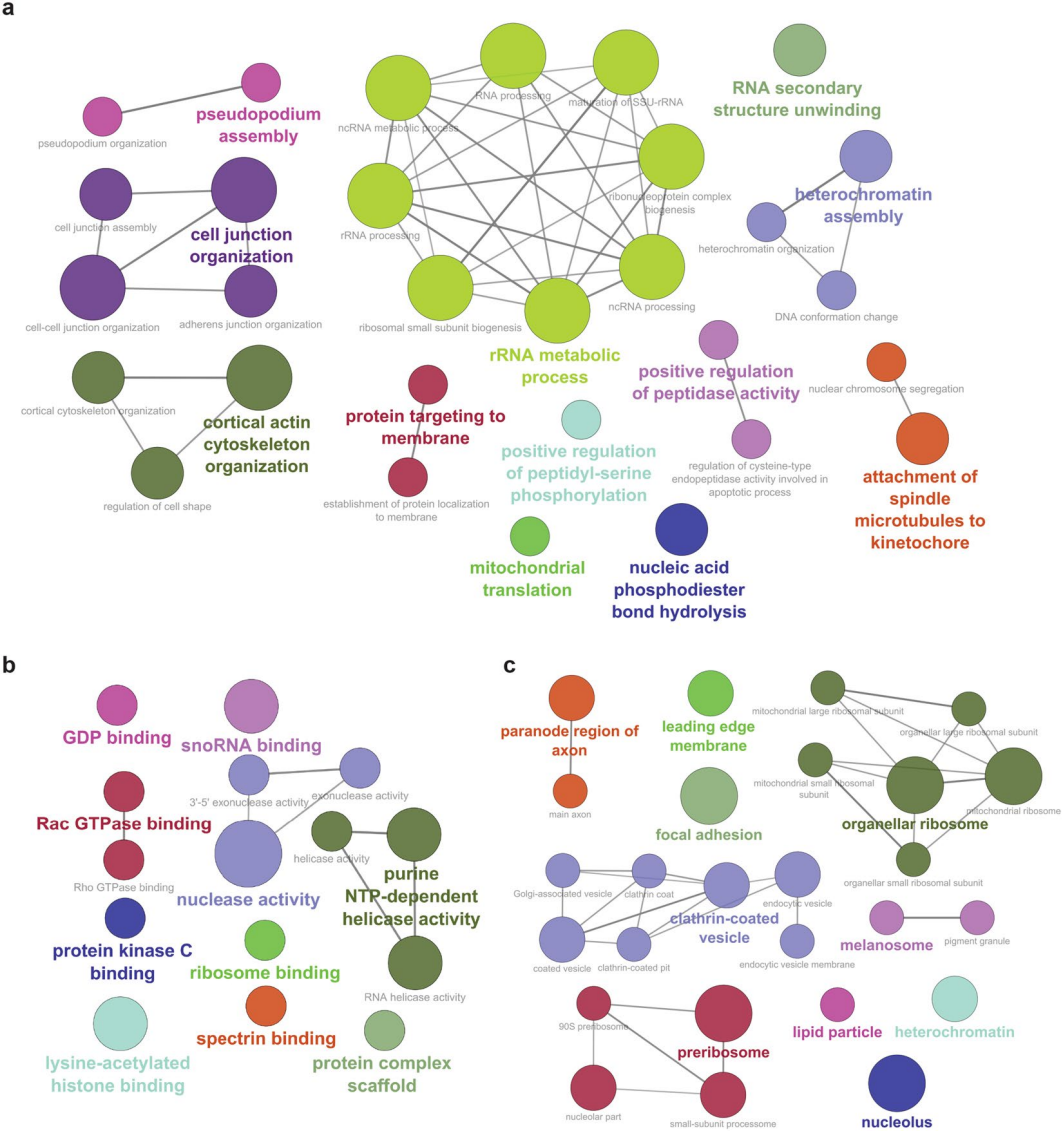
GO biological processes, molecular functions and cellular components represented by candidate MARCKS interactors. (**a**) GO biological processes enriched in the combined MARCKS BioID dataset. (**b**) GO molecular functions enriched in the combined MARCKS BioID dataset. (**c**) GO cellular components enriched in the combined MARCKS BioID dataset. Weight of solid lines reflects number of shared genes found among pathways. Larger node size reflects increased significance, with p < 0.05 for all nodes.

To assign functional significance to our candidate interactors, we used publicly available Gene Ontology Consortium (GO) data to ask what biological processes, molecular pathways and cellular compartments were represented in our data. In addition to confirming well-validated roles for MARCKS in regulation of the actin cytoskeleton, we identified statistically significant enrichment for a number of GO biological processes related to neurite morphogenesis, including “regulation of cell shape”, “protein targeting to the membrane” and “pseudopodium assembly and organization” (Fig. 4a). Similarly, in addition to validating a molecular role as a “protein kinase C binding” protein, we identified significant enrichment for a number of other GO molecular functions which are critical for neurite dynamics, including “Rac GTPase binding”, “Rho GTPase binding” and “GDP binding” (Fig. 4b). Our dataset was enriched for several GO cellular components, including “main axon”, “leading edge membrane” and “focal adhesion”, all areas involved in neurite outgrowth (Fig. 4c). Together, these results strongly suggest that MARCKS could participate in the dynamic regulation of cell shape within neuronal processes, potentially by modulating small GTPases like RAC, RHO and CDC42.

**Figure 5:**
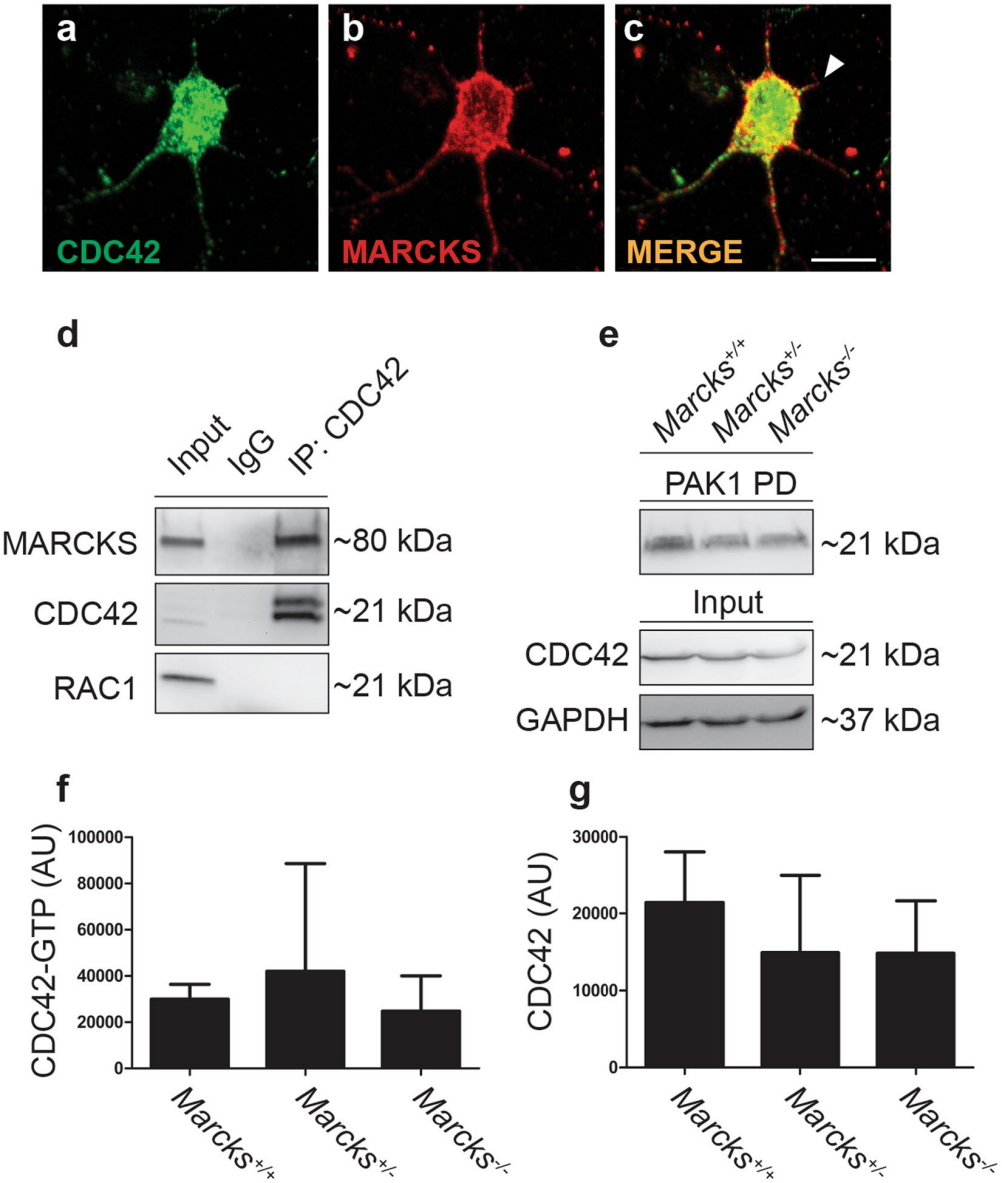
MARCKS interacts with CDC42, but does not influence whole brain levels of CDC42 activation. (**a**–**c**) CDC42 (green, **a**) colocalizes with MARCKS (red, **b**) in developing neurites (white arrowhead, **c**). (**d**) CDC42 coimmunoprecipitates with MARCKS from E18.5 mouse whole-brain lysates. (**e**–**g**) Levels of GTP-bound (active) CDC42 (PAK1 PD) are not significantly different in E18.5 brain lysates from *Marcks*^+/+^, *Marcks*^−/+^ and *Marcks*^*−/−*^ embryos. Lysates (input, separate gel) blotted for total CDC42 and GAPDH serve as loading controls. (**f**,**g**) Quantification of CDC42 activity assay shown in (**e**). One-way ANOVA, *p < 0.05 level using post-hoc Tukey’s correction for multiple comparisons. n = 3/genotype. Bar graphs are mean ± SEM. Scale bar in (**c**) is 10 μm.

The interactors identified in complex 1 have well established roles in neurite initiation, outgrowth, navigation and branching. We validated the interaction with CDC42 with coimmunoprecipitation from E18.5 mouse brain lysates (Fig. 5d) to confirm that this interaction occurs *in vivo*. To establish that MARCKS and CDC42 interactions occur early on in neurite outgrowth we examined CDC42 and MARCKS localization in 2 DIV primary mouse neurons and observed co-localization of the two proteins in nascent neurites (Fig. 5a–c**)** Co-localization was also observed in primary mouse fibroblasts (Supplemental Fig. 2). Since CDC42 GTP-binding and CDC42 activity is regulated by localization to the inner leaflet of the plasma membrane^24^, we hypothesized that MARCKS could be regulating this process. To examine this possibility, we performed CDC42 activity assays to quantify GTP-bound CDC42 from E18.5 brain lysates from *Marcks*^+/+^, *Marcks*^−/+^ and *Marcks*^*−/−*^ embryos. Total levels of CDC42, as well as levels of activated CDC42, were not significantly altered in *Marcks*^*−/−*^ brains (Fig. 5e–g). This suggests that if MARCKS is regulating CDC42 activation, it must be occurring in specific microdomains, rather than in a manner which would measurably affect whole cell levels of activated CDC42.

## Discussion

The work presented here demonstrates for the first time that MARCKS regulates neurite initiation in cortical neurons. Furthermore, we show that MARCKS interacts with a host of cellular machinery that regulates cell shape and process extension through modulation of the cytoskeleton. These interactors include CDC42, ARF1, SRGAP2, PAK4 and other proteins which have all been implicated in modulating actin polymerization and neurite dynamics. While overall levels of CDC42 activity in embryonic brains are not changed in the absence of MARCKS, there are a variety of mechanisms by which MARCKS could still be influencing CDC42-related activities.

In radial glial progenitors, MARCKS regulates the apical localization of CDC42, along with a number of other proteins critical for radial glia polarity^7^. Therefore, one possibility is that in neurons, MARCKS could similarly control the localization of CDC42 and its regulators and effectors. This could be through direct interactions, or through the modulation of lipid-coordinated signaling domains at the inner leaflet of the plasma membrane. MARCKS is a critical regulator of PIP2 levels in a variety of cell types, including neurons and PIP2 interactions regulate the membrane targeting of a variety of signaling proteins, including CDC42 and other small GTPases^21,25–27^. In epithelial cells, eliminating apical PIP2 clustering disrupts CDC42 localization and CDC42-dependent morphogenesis^28^. Examining the subcellular localization patterns of PIP2, MARCKS and downstream interactors in developing neurites may therefore reveal some of these relationships in neurons.

Our GO analysis highlighted roles for MARCKS in specific subcellular locales within developing processes, including in the lamellipodia of the leading edge and at focal adhesions. While true focal adhesions are absent in neurons, they have many similarities to the integrin-containing neuronal point contacts that are critical for neurite morphogenesis. Previous studies have also suggested that MARCKS could be regulating cytoskeletal modulation through integrins and focal adhesion proteins^9,29^. We recently identified an important role for MARCKS in the regulation of focal adhesion kinase (FAK) localization in multiple cell types, including neurons^9^. FAK, a membrane-associated tyrosine kinase, serves as a critical integrator of multiple signals from growth factor and adhesion receptors^30–33^. While FAK was not identified in this *in vitro* BioID dataset, FAK has been shown to directly regulate CDC42 activity in neuronal growth cones^34^ and FAK is known to be an important player in neuritogenesis^35^. As such, it is likely that MARCKS is influencing neurite initiation through interactions with CDC42, FAK and other key partners.

While we identified a role for MARCKS in neurite initiation, more work will be required to pinpoint precisely where and when MARCKS is needed for this process. Multiple modes of early neuritogenesis have been described. In some cases, it appears that filopodia develop directly from the neuronal cell body and are then stabilized as nascent neurites^36,37^. In other cases, large lamellipodia develop and extend circumferentially and then collapse and condense to form neurites^37,38^. More detailed examination of MARCKS function at these early stages of neurite initiation should clarify precisely where MARCKS is operating. Furthermore, while we examined neuritogenesis in primary cortical neurons, modes of neurite initiation appear to vary depending on the neuronal subtype^14^ and it remains to be seen whether MARCKS is universally required for this process.

In recent years, multiple lines of evidence have suggested that MARCKS is a critical regulator of membrane-localized signaling networks that regulate cytoskeletal dynamics and cell shape^9,11,25,29,39–41^. As more studies shed light on the mechanism by which MARCKS influences these processes, new insights will be gained into how highly dynamic cells, like neurons, remodel their membranes as they differentiate. Understanding how these signaling networks are organized and coordinated may also help us better understand how these processes are perturbed in disease states, where modulation of targets including MARCKS could prove therapeutically valuable.

## Methods

### Mice

Animal protocols were approved by the Sanford Research Institutional Animal Care and Use Committee (USDA License 46-R-0009) with all procedures carried out in strict accordance with National Institutes of Health (NIH) guidelines and the Sanford Research Institutional Animal Care and Use Committee guidelines. All mice were maintained as heterozygotes on a C57BL/6 J background. *Marcks*^+/−^ mice^42^ were obtained from Dr. Perry Blackshear at the Signal Transduction Laboratory of the NIH Intramural Research Program (Research Triangle Park, NC USA).

### Neuronal Morphology Analysis

Primary neuronal cultures were isolated from embryonic day 15.5 (E15.5) mice, plated on glass cover slips and maintained as previously described^43^. At 5DIV, cultures were fixed for 20 minutes with ice-cold 2% paraformaldehyde, 4% sucrose in phosphate buffered saline (PBS). Cultures were rinsed 3x in PBS and stored in PBS with 0.02% sodium azide at 4 °C. Immunocytochemistry was performed as previously described^9^, with primary antibodies (1:1000) directed against TUBB3 (Aves Labs, Tigard, OR) to label neuronal arbors. Neurons were imaged using a Nikon Eclipse Ni-E upright microscope using a 20x dry objective lens. Neurons were traced using Neurolucida software (MBF Bioscience) and neurite counts and Sholl analysis were conducted using Neurolucida Explorer software.

### High Content Screening analysis of neurite outgrowth

Primary neuronal cultures were collected and processed as described above. 100 uM of MANS peptide, diluted in media, was administered at either 12 hours, 72 hours, or 12 and 72 hours after initial plating. Neurite screening was performed using a CellInsight CX7 High Content System, using the Neurite BioApplication (Version 4, Thermofisher). 150 neurons were analyzed from each well and the average values from each well were reported (n = 8–12 wells per condition). Statistical significance was determined with either a Student’s T-test, or a 1-way ANOVA followed with a Tukey’s Post Hoc test.

### Cloning, Plasmids, and Viral Transduction

The retroviral expression constructs for MARCKS-BirA and BirA control were created by cloning the MARCKS-BirA or BirA transgenes from pMARCKS-BioID^9^ into the EcoRI site of pBABE-puro using In Fusion Cloning (Clontech, Mountain View, CA). Retrovirus was produced using the amphotropic Phoenix retroviral packaging system (ATCC# CRL-3213) and N2A cells (ATCC# CCL-131) were transduced as previously described^19,44^. Transduced cells were selected with puromycin (20 μg/ml) and minimally passaged prior to expansion and drug-treatment for BioID studies.

### MARCKS BioID in N2A Cells

Stable cell lines expressing MARCKS-BirA and BirA only were maintained in DMEM with 10% fetal bovine serum (FBS) and 2 μg/ml puromycin. For differentiation, when cells reached approximately 50% confluency, serum was reduced to 2% and 20 μM retinoic acid (R2625, Sigma) or 1 mM db-cAMP (SC201567, Santa Cruz) were added in the media for 7 days. 50 μM biotin was added to the media 24 hours prior to collection. Cells were lysed and biotinylated proteins were captured using immobilized streptavidin as previously described^45^. Following capture, proteins were sent to the Sanford Burnham Prebys Proteomics core for mass spectrometry. Captured proteins were digested directly on-beads. Briefly, proteins bound to the beads were resuspended with 8 M urea, 50 mM ammonium bicarbonate and cysteine disulfide bonds were reduced with 10 mM tris(2-carboxyethyl)phosphine (TCEP) at 30 °C for 60 min followed by cysteine alkylation with 30 mM iodoacetamide (IAA) in the dark at room temperature for 30 min. Following alkylation, urea was diluted to 1 M urea using 50 mM ammonium bicarbonate and proteins were subjected to overnight digestion with mass spec grade Trypsin/Lys-C mix (Promega, Madison, WI). Peptide-containing supernatant was collected and beads were then washed once with 50 mM ammonium bicarbonate to increase peptide recovery. The digested samples were desalted using a C18 TopTip (PolyLC, Columbia, MD) and the organic solvent was removed in a SpeedVac concentrator prior to LC-MS/MS analysis.

### LC-MS/MS Analysis

Dried samples were reconstituted with 2% acetonitrile, 0.1% formic acid and analyzed by LC-MS/MS using a Proxeon EASY nanoLC system (Thermo Fisher Scientific) coupled to an Orbitrap Elite mass spectrometer (Thermo Fisher Scientific). Peptides were separated using an analytical C18 Acclaim PepMap column 0.075 × 250 mm, 2 µm particles (Thermo Scientific) in a 180-min gradient of 2–28% solvent B at a flow rate of 300 nL/min. The mass spectrometer was operated in positive data-dependent acquisition mode. MS1 spectra were measured with a resolution of 60,000, an AGC target of 1e6 and a mass range from 350 to 1400 m/z. Up to 10 MS2 spectra per duty cycle were triggered, fragmented by collision-induced dissociation and acquired in the ion trap with an AGC target of 1e4, an isolation window of 2.0 m/z and a normalized collision energy of 35. Dynamic exclusion was enabled with duration of 30 sec.

### Mass Spec Data Analysis

All mass spectra were analyzed with MaxQuant software version 1.5.5.1. MS/MS spectra were searched against the *M. musculus* Uniprot protein sequence database (version July 2016) and GPM cRAP sequences (commonly known protein contaminants). Precursor mass tolerance was set to 20 ppm and 4.5 ppm for the first search where initial mass recalibration was completed and for the main search, respectively. Product ions were searched with a mass tolerance 0.5 Da. The maximum precursor ion charge state used for searching was 7. Carbamidomethylation of cysteines was searched as a fixed modification, while oxidation of methionines and acetylation of protein N-terminal were searched as variable modifications. Enzyme was set to trypsin in a specific mode and a maximum of two missed cleavages was allowed for searching. The target-decoy-based false discovery rate (FDR) filter for spectrum and protein identification was set to 1%.

### Network Analysis

BioID candidate interactors were identified as proteins present in MARCKS-BirA cell lines at levels at least 3-fold greater than in BirA-only controls. Each condition (undifferentiated, RA, db-cAMP) was compared to the corresponding control condition. Common contaminants^46^ were manually removed from the dataset. Network maps were created using Cytoscape 3.4.0, with the ClueGO^47^ and PEWCC 1.0^23^ plugins. STRING maps were created using a confidence score of 0.6 and maximum number of interactors set at “0”. GO network maps were created using all evidence codes, medium network specificity, GO term fusion and a p-value cutoff of 0.05. Noise filtering and weighted clustering was performed with PEWCC, using a join parameter of 0.3 and an overlap threshold of 0.5.

### Immunoprecipitation and Western Blotting

MARCKS immunoprecipitation and Western blots were performed as previously described^9^. Briefly, MARCKS was immunoprecipitated using the Pierce Crosslink IP kit (Thermo Fisher) according to the supplied protocol, with crosslinking steps omitted and 10 μg of anti-MARCKS antibodies (SC6455, Santa Cruz) conjugated to protein A/G agarose beads. Active CDC42 was isolated as previously described^48^, using an immobilized GST fusion construct of the CDC42 binding domain of mouse p65^PAK^, kindly provided by K. Burridge & Lisa Sharek. Western Blots were performed using 0.45 µm pore Immobilon PVDF membranes (Thermo Fisher) and using 2.5% nonfat dried milk in tris-buffered saline with 0.1% Tween-20 for blocking and antibody incubation steps. Primary antibodies and HRP-conjugated probes included anti-MARCKS (Santa Cruz SC6455, 1:1000), anti-CDC42 (BD Biosciences 610928, 1:1000), anti-BirA (Abcam 14002, 1:3000), anti-Rac1 (Cell Signaling 2465, 1:1000), anti-GAPDH (Cell Signaling 5174, 1:3000) and Streptavidin-HRP (Thermo Fisher N100, 1:40,000).

### Statistics

A minimum of three technical replicates were performed for neuronal culture experiments. All quantifications are reported as the mean plus or minus the standard error in the mean (error bars). Statistical analysis was performed in Graphpad Prism 5.02. Student’s t-tests were performed as two-tailed tests, with F-tests used to compare variances and p < 0.05 used as the cutoff for significance. One-way ANOVAs were performed with post-hoc t-tests with Tukey’s multiple comparison correction comparing all possible groups.

## Electronic supplementary material

Supplementary Materials

## Data Availability

The datasets generated during and/or analyzed during the current study are available from the corresponding author on reasonable request.
