# Supplementary material for: MARCKS regulates neuritogenesis and interacts with a CDC42 signaling network
Source: Sci Rep. 2018 Sep 5;8:13278. doi: 10.1038/s41598-018-31578-0 (PMC6125478; doi:10.1038/s41598-018-31578-0)
Supplement: Supplementary file 1 — Supplementary Materials [file 41598_2018_31578_MOESM1_ESM.pdf]

SUPPLEMENTARY MATERIALS for

MARCKS regulates neuritogenesis and interacts with a CDC42 signaling network.

Brudvig JJ, Cain JT, Sears RM, Schmidt-Grimminger GG, Wittchen ES, Ghashghaei HT, Adler KB, Weimer JM*

*Corresponding Author. Jill.Weimer@sanfordhealth.org. (605) 312-6407.

```
MARCKS (ED), aa 140-182   SSETPKKKKKRFSFKKSFKLSGFSFKKSKKESGEGAEAEGATA
ADD3, aa 678-706          PSKSP-SKKK----KK-FRTPSF-LKKNKKK--EKVEA
BASP1, aa 7-36                  KKKK-GYNVNDEKAKD----KDKKAEGAGTEEEG-TP
RALBP1, aa 114-156        KPSFSKKKEKDFKIKEKPKEEKHKEEKHKEEKHKEKKSKDLTA

MARCKS (ED), aa 140-182   SSETPKKKKKRFSFKKSFKLSGFSF--KKSKKE-SG--E--GA---EAEGATA
AKAP12, aa  141-193                     KKVFKFVGFKFTVKKDKNEKSDTVQLLTVKKDEGEGAEA
```

Supplemental Figure 1. Proteins expressed in brain with domains homologous to the MARCKS ED-domain.

Amino acid sequence alignments between selected *M. musculus* proteins and the MARCKS ED. Shared residues are in red.

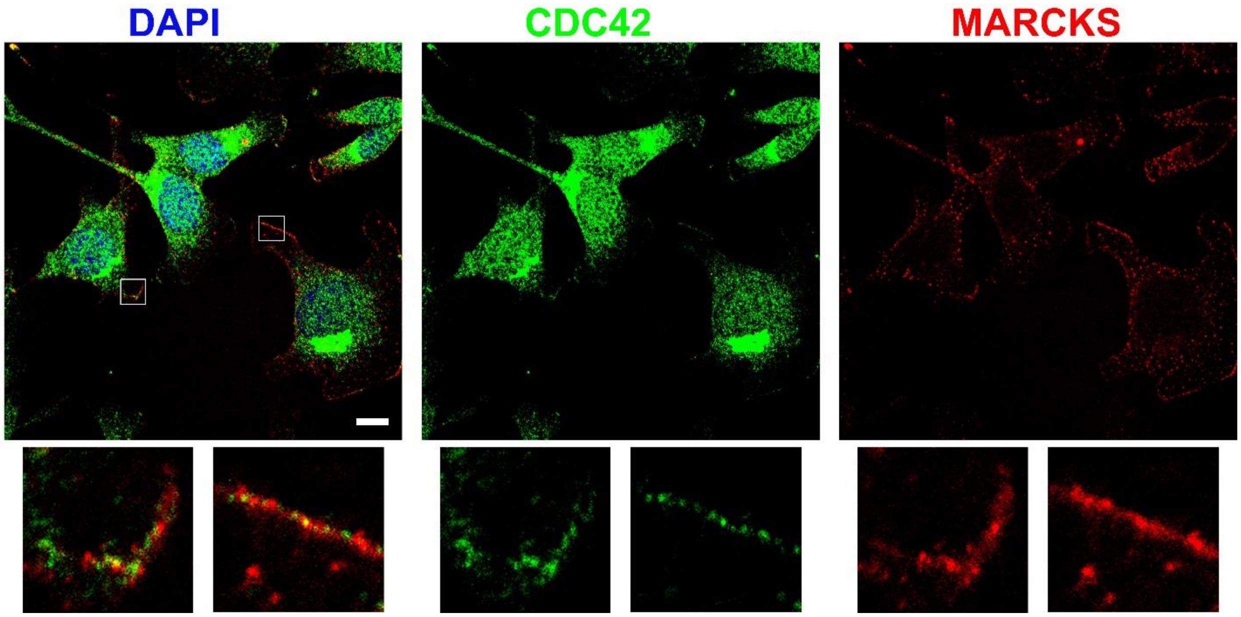

Supplemental Figure 2. MARCKS and CDC42 co-localize at the edge of the plasma membrane.

Primary mouse fibroblasts fluorescently labeled with CDC42 (green), MARCKS (red), and DAPI (blue) and imaged at 100x (scalebar = 10um), show colocalization at the plasma membrane.

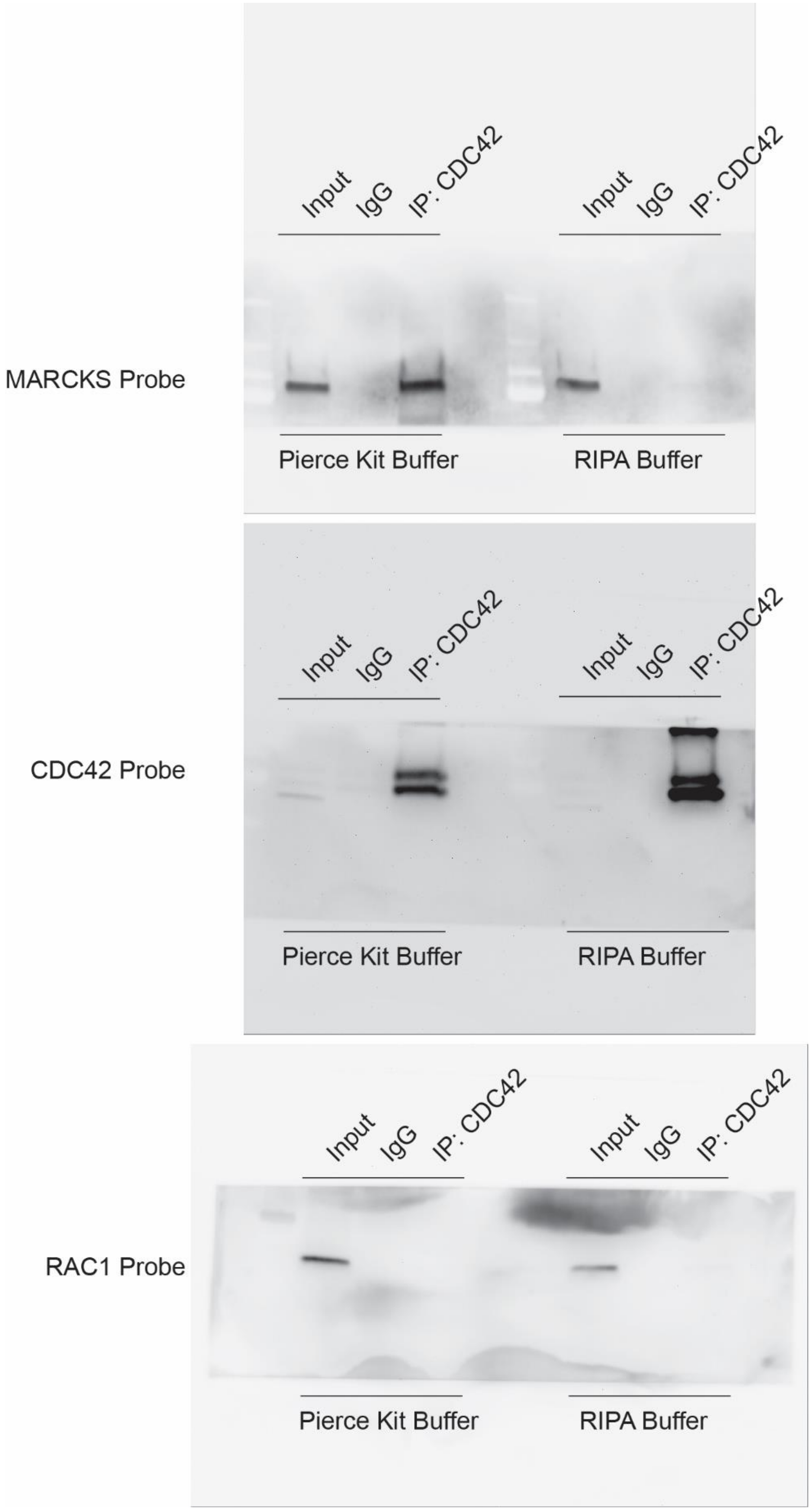

Uncropped Western Blot Images from Figure 5d

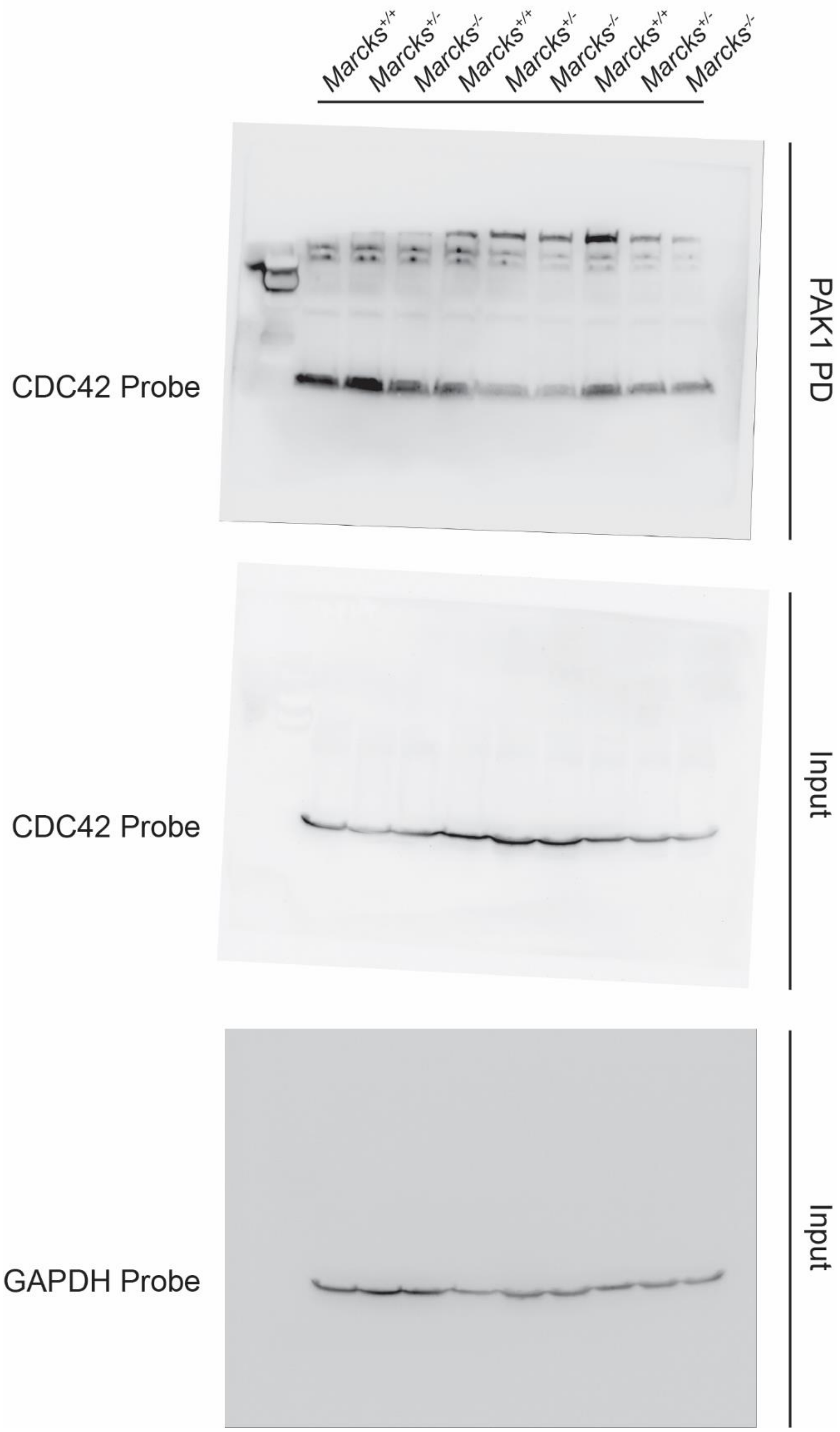

Uncropped Western Blot Images from Figure 5e
